# Bactericidal activities and post-antibiotic effects of ofloxacin and ceftriaxone against drug-resistant *Salmonella enterica* serovar Typhi

**DOI:** 10.1093/jac/dkab215

**Published:** 2021-06-28

**Authors:** John Wain, Julie A Simpson, Luong Thi Diem Nga, To Song Diep, Pham Thanh Duy, Stephen Baker, Nicholas P J Day, Nicholas J White, Christopher M Parry

**Affiliations:** 1 Wellcome Trust Major Overseas Programme, Oxford University Clinical Research Unit, Hospital for Tropical Diseases, Vo Van Kiet, District 5, Ho Chi Minh City, Vietnam; 2 Quadram Institute Bioscience, Norwich Research Park, Norwich, UK; 3 Centre for Epidemiology and Biostatistics, Melbourne School of Population and Global Health, University of Melbourne, Melbourne, Australia; 4 Mahidol-Oxford Tropical Medicine Research Unit, Faculty of Tropical Medicine, Mahidol University, Bangkok, Thailand; 5 Hospital for Tropical Diseases, Vo Van Kiet, District 5, Ho Chi Minh City, Vietnam; 6 Centre for Tropical Medicine and Global Health, Nuffield Department of Clinical Medicine, University of Oxford, Oxford, UK; 7 Department of Medicine, University of Cambridge, Cambridge, UK

## Abstract

**Background:**

The clinical response to ceftriaxone in patients with typhoid fever is significantly slower than with ofloxacin, despite infection with *Salmonella enterica* serovar Typhi (*S.* Typhi) isolates with similar susceptibilities (MIC 0.03–0.12 mg/L). The response to ofloxacin is slower if the isolate has intermediate susceptibility (MIC 0.25–1.0 mg/L).

**Objectives:**

To determine the bactericidal activity and post-antibiotic effect (PAE) of ceftriaxone and ofloxacin against *S.* Typhi.

**Methods:**

The mean time to reach a 99.9% reduction in log_10_ count (bactericidal activity) and PAE of ceftriaxone and ofloxacin were determined for 18 clinical isolates of *S.* Typhi in time–kill experiments (MIC range for ofloxacin 0.06–1.0 mg/L and for ceftriaxone 0.03–0.12 mg/L).

**Results:**

The mean (SD) bactericidal activity of ofloxacin was 33.1 (15.2) min and 384.4 (60) min for ceftriaxone. After a 30 min exposure to ofloxacin, the mean (SD) duration of PAE was 154.7 (52.6) min. There was no detectable PAE after 1 h of exposure to ceftriaxone. For ofloxacin, bactericidal activity and PAE did not significantly differ between isolates with full or intermediate susceptibility provided ofloxacin concentrations were maintained at 4×MIC.

**Conclusions:**

Infections with *S.* Typhi with intermediate ofloxacin susceptibility may respond to doses that maintain ofloxacin concentrations at 4×MIC at the site of infection. The slow bactericidal activity of ceftriaxone and absent PAE may explain the slow clinical response in typhoid.

## Introduction

Typhoid (enteric) fever, caused by *Salmonella enterica* serovar Typhi (*S.* Typhi) and serovar Paratyphi A (*S.* Paratyphi A), causes morbidity and mortality in children and young adults in low- and middle-income countries where adequate sanitation and clean water are lacking.^1^ Effective antimicrobial therapy shortens illness duration and reduces complications and mortality, but resistance to commonly used agents is widespread.^1^ A recent systematic review reports a pooled prevalence (95% CI) of fluoroquinolone-non-susceptible *S.* Typhi in South Asia between 2015 and 2018 of 70% (38%–94%).^2^ A large outbreak of ciprofloxacin- and ceftriaxone-resistant typhoid has affected the Sindh province in Pakistan since 2016.^3^ The *S.* Typhi clade H58 has been particularly dominant in the spread of these resistant strains across Asia and some parts of Africa.^4^

Typhoid fever treatment with ofloxacin results in rapid recovery times and high cure rates even with short treatment courses, provided the infecting isolates have an MIC ≤0.1 mg/L.^5–9^ Infections with isolates with intermediate susceptibility to ofloxacin, defined by an ofloxacin MIC of 0.25–1.0 mg/L, or resistance to nalidixic acid or pefloxacin, have prolonged recovery times and increased clinical failure rates.^10–12^ Ceftriaxone has an MIC of ≤0.1 mg/L, in a similar range to susceptible fluoroquinolones, but, when used for treatment, the fever recovery times are slow.^5^^,^^13–15^

We compared the *in vitro* bactericidal activities and the post-antibiotic effects (PAEs) of ofloxacin and ceftriaxone against clinical isolates of *S*. Typhi.

## Materials and methods

### Bacterial strains and patients

The study used 18 unique blood culture isolates of *S.* Typhi from Vietnamese patients with uncomplicated typhoid fever before entry into randomized controlled trials of treatment with short courses of ofloxacin (Oflocet^®^; Roussel-UCLAF, France) reported previously.^6^^,^^7^ The response to treatment was assessed by fever clearance time, defined as the time since treatment began for the temperature to fall below 37.5°C and remain at or below 37.5°C for 48 h.

Strains were identified by standard biochemical tests and by agglutination with specific antisera (Murex, Dartford, UK). Antimicrobial susceptibility testing against chloramphenicol (30 μg), ampicillin (10 μg), co-trimoxazole (1.25/23.75 μg), ceftriaxone (30 μg) and nalidixic acid (30 μg) discs was performed and interpreted using CLSI guidelines.^8^ The MIC and MBC of ofloxacin (Roussel-UCLAF, France) and ceftriaxone (Rocephin^®^; Roche, Hong Kong, China) were determined by the microdilution method with CAMHB (Difco, MI, USA).

### Molecular typing

We determined by modified pyrosequencing if the serovar Typhi strains were the H58 clade, by inferring genotype though the detection of the H58-specific SNP and the common SNPs located at positions 83 and 87 in the *gyrA* gene and position 80 in the *parC* that determine intermediate susceptibility to ofloxacin in the isolates resistant to nalidixic acid.^16^

### Time–kill studies

Ten mL of warm Mueller–Hinton broth containing either ofloxacin (Roussel-UCLAF, France) or ceftriaxone (Rocephin^®^; Roche, Hong Kong, China) at a concentration of 8×MIC for the strain used in that experiment was added at time zero to 10 mL of warm Mueller–Hinton broth containing the isolate at a bacterial density of approximately 2 × 10^6^ cfu/mL in log-phase growth to give a final concentration of drug in the broth of 4×MIC. In the controls, the added broth contained no drug. The cultures were incubated with shaking at 100 rpm at 35–37°C for 24 h. Samples for viable counts were taken immediately before the addition of the drug or control broth and then at 0.25, 0.5, 1, 2, 4, 6, 9, 12 and 24 h after the addition of drug. Viable counts were performed in duplicate [see the Supplementary Methods (available as Supplementary data at *JAC* Online)]. Each experiment was performed in triplicate.

### PAE

For determination of the PAE, 200 μL of broth was removed from each of the time–kill broths at exactly 30 min (ofloxacin) and 1 h (ceftriaxone) after the addition of drug and diluted to 1/100 in 20 mL of warm Mueller–Hinton broth. For the controls, 200 μL of the time–kill control broth was removed and added to 20 mL of warm Mueller–Hinton broth containing the same final concentration of antimicrobial agent as in the test PAE tube. Viable counts were performed immediately after dilution and at hourly intervals for at least 6 h.

### Determination of bactericidal activity and PAE

Log viable counts were plotted versus time. The bactericidal activity was the time to reach a 99.9% reduction in log_10_ count and was calculated by two people with differences reconciled. The PAE was calculated as the PAE = T − C, where C is the time for a 1 log_10_ increase in the viable count from the moment of dilution of the PAE control and T is the time for a 1 log_10_ increase from the time of dilution for the test. The mean (SD) for the bactericidal activity and PAE for each resistance group was compared using one-way analysis of variance.

## Results

### MICs and MBCs, haplotype and gyrA mutations

Six isolates were fully susceptible to the drugs tested (FS: susceptible to chloramphenicol, ampicillin, co-trimoxazole, ceftriaxone and nalidixic acid, and with an ofloxacin MIC ≤0.12 mg/L), six were MDR NA^S^ (resistant to chloramphenicol, ampicillin and co-trimoxazole, but susceptible to ceftriaxone and nalidixic acid, and with an ofloxacin MIC ≤0.12 mg/L) and six were MDR NA^R^ (resistant to chloramphenicol, ampicillin, co-trimoxazole and nalidixic acid, but susceptible to ceftriaxone, and with an ofloxacin MIC of 0.25–1.0 mg/L). One MDR NA^R^ strain had an ofloxacin MIC of 0.12 mg/L. The response of patients infected with isolates in each resistance group to ofloxacin treatment is shown in Table S1 and Figure S1 (both available as Supplementary data at *JAC* Online). The fever clearance time was prolonged (>200 h) for five of the six patients infected with an MDR NA^R^ isolate; three patients required rescue antimicrobial treatment, including one patient with a repeat positive blood culture who also developed gastrointestinal bleeding. Six isolates were haplotype H58 and 10 were non-H58. A single point mutation in the QRDR of the *gyrA* gene at D87G (GAC→GGC) was found in four of the MDR NA^R^ isolates and S83F (TCC→TTC) in one MDR NA^R^ isolate. The remaining MDR NA^R^ isolate was unavailable for typing.

### Time–kill studies and PAE

**Figure 1.**
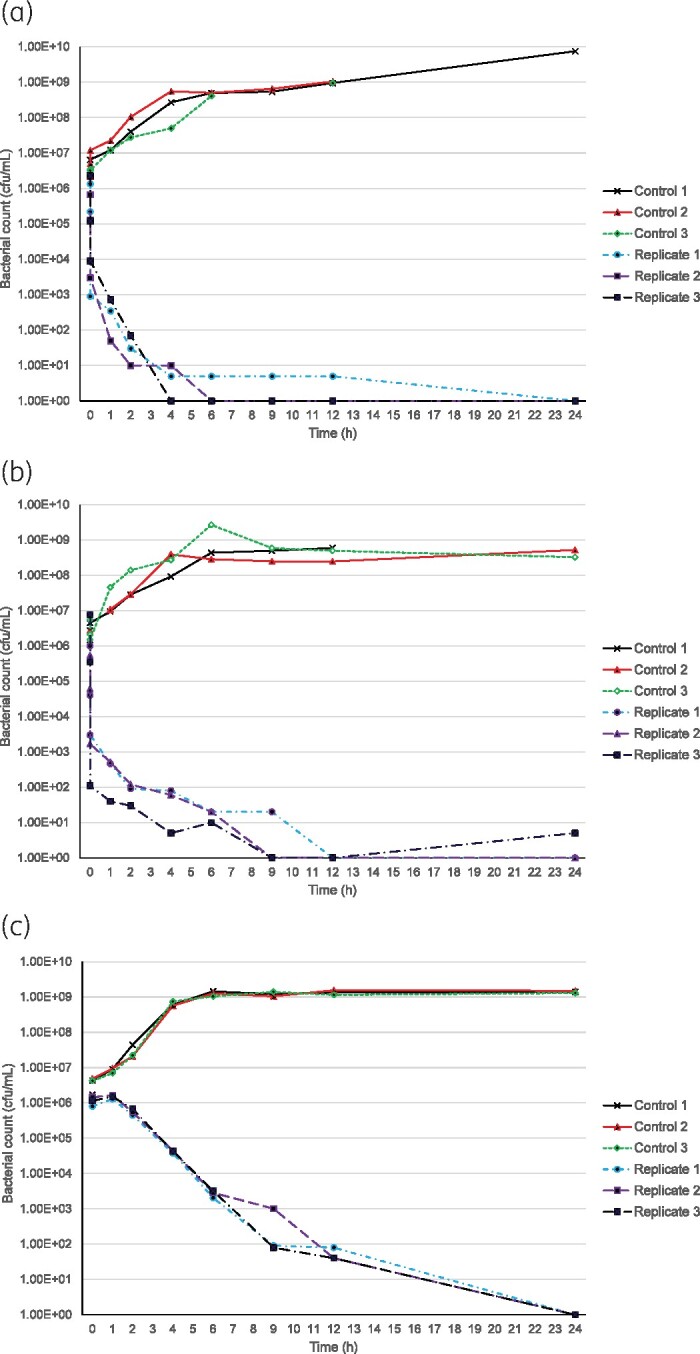
(a) Time–kill curve for isolate from patient CT65, an MDR NA^S^*S.* Typhi isolate (ofloxacin MIC 0.06 mg/L) exposed to ofloxacin at 4×MIC. (b) Time–kill curve for isolate from patient TY169, an MDR NA^R^*S.* Typhi isolate (ofloxacin MIC 1.0 mg/L) exposed to ofloxacin at 4×MIC. (c) Time–kill curve for isolate from patient CT55, an MDR NA^S^*S.* Typhi isolate (ceftriaxone MIC 0.12 mg/L) exposed to ceftriaxone at 4×MIC. This figure appears in colour in the online version of *JAC* and in black and white in the print version of *JAC*.

The mean (SD) bactericidal activity of ofloxacin in the 18 experiments was 33.1 (15.2) min, compared with 384.4 (60) min for ceftriaxone (Table 1). Typical curves are shown in Figure 1. After 30 min of exposure to ofloxacin, all isolates gave a PAE; mean (SD) PAE of 157.7 (11.2) min. There were no significant differences for the time–kill and PAE when comparing the FS, MDR NA^S^ and MDR NA^R^ isolates exposed to ofloxacin at 4×MIC. There was no detectable PAE after 1 h of exposure to ceftriaxone.

**Table 1.** Pharmacodynamic parameters for 18 isolates of *S.* Typhi versus ofloxacin and ceftriaxone [values are the median (range) of three separate experiments and the mean (SD) for all patient data combined]

Patient code	Susceptibility pattern^a^	Ofloxacin		Ceftriaxone
		time–kill (min)	PAE (min)	time–kill (min)
TY65	FS	35 (25–45)	125 (125–215)	320 (155–450)
TY73	FS	25 (25–25)	190 (185–245)	315 (305–450)
TY86	FS	20 (20–20)	240 (85–255)	280 (265–330)
TY98	FS	25 (25–25)	60 (165–165)	475 (192–475)
CT4	FS	35 (35–70)	155 (145–165)	425 (350–435)
CT66	FS	50 (50–50)	110 (30–125)	455 (455–455)
**All FS [mean (SD)]**		**31.7 (10.8)**	**146.7 (63.2)**	**378.3 (83.0)**
TY77	MDR NA^S^	25 (25–25)	215 (210–285)	385 (255–475)
TY84	MDR NA^S^	40 (40–40)	160 (135–185)	440 (415–490)
TY90	MDR NA^S^	16 (16–16)	200 (135–300)	405 (385–415)
TY97	MDR NA^S^	35 (21–66)	180 (125–280)	315 (280–330)
CT55	MDR NA^S^	42 (30–50)	100 (65–190)	415 (395–495)
CT65	MDR NA^S^	70 (30–100)	100 (50–160)	330 (330–345)
**All MDR NA^S^ [mean (SD)]**		**38.0 (18.5)**	**159.2 (49.4)**	**381.7 (49.4)**
TY62	MDR NA^R^	55 (49–55)	110 (85–115)	435 (420–450)
TY169	MDR NA^R^	45 (20–75)	125 (105–180)	420 (355–445)
CT30	MDR NA^R^	23 (18–25)	175 (60–190)	425 (420–605)
CT31	MDR NA^R^	25 (25–25)	105 (25–130)	425 (325–425)
CT75	MDR NA^R^	10 (10–40)	200 (105–270)	345 (320–450)
CT76	MDR NA^R^	20 (20–95)	235 (205–235)	310 (300–325)
**All MDR NA^R^ [mean (SD)]**		**29.7 (16.9)**	**158.3 (53.3)**	**393.3 (52.4)**

a FS, susceptible to chloramphenicol, ampicillin, co-trimoxazole, ceftriaxone and nalidixic acid, and with an ofloxacin MIC ≤0.12 mg/L; MDR NA^S^, resistant to chloramphenicol, ampicillin and co-trimoxazole, but susceptible to ceftriaxone and nalidixic acid, and with an ofloxacin MIC ≤0.12 mg/L; MDR NA^R^, resistant to chloramphenicol, ampicillin, co-trimoxazole and nalidixic acid, but susceptible to ceftriaxone, and with an ofloxacin MIC of 0.25–1.0 mg/L.

## Discussion

Ofloxacin at a concentration of 4×MIC was rapidly bactericidal against susceptible and MDR strains of *S.* Typhi that had ofloxacin MICs ≤0.12 mg/L and those with an MIC of 0.25–1.0 mg/L. Ofloxacin also provided an average PAE of 158 min against these organisms. In contrast, ceftriaxone at four times the MIC of ≤0.12 mg/L demonstrated a slow bactericidal action against all strains and no PAE.

A pharmacokinetic-pharmacodynamic measure that correlates with *in vivo* efficacy of ofloxacin in typhoid is thought to be *fC*_max_/MIC.^17^ In Vietnamese children with uncomplicated typhoid fever, treated with 15 mg/kg/day of oral ofloxacin in two divided doses, the mean (95% CI) peak serum level was 5.5 (4.7–6.3) mg/L.^18^ Ofloxacin is approximately 35% bound to plasma proteins, so this corresponds with an unbound peak level of approximately 3.6 mg/L. In adult healthy volunteers given 200 mg of ofloxacin, a dose used in a number of the clinical trials, the serum level at 2 h was 2.07 mg/L with an estimated unbound concentration of 1.3 mg/L.^19^ For isolates of *S*. Typhi with an MIC of ≤0.06 mg/L the *fC*_max_/MIC ratio will therefore vary between 60:1 and 22:1, well above the 4×MIC. For isolates with intermediate ofloxacin susceptibility the *fC*_max_/MIC ratio will range between 14.4:1 and 3.6:1 in children and between 5.2:1 and 1.3:1 in adults. Although isolates with intermediate susceptibility also demonstrated a PAE, the unbound serum level will fall below a value of 4×MIC during the dosing cycle.

Ceftriaxone had a slow bactericidal action against all strains and no PAE against all the *S.* Typhi. A pharmacokinetic-pharmacodynamic target associated with treatment success of ceftriaxone against other Gram-negative infections is a target attainment of drug levels above the MIC for 100% of the dosing cycle. The mean (SD) peak serum ceftriaxone level in adolescents and adults with typhoid in Nepal, treated with 3 g of IV ceftriaxone once daily, was 291 (92) mg/L and the trough was 21.7 (25.4) mg/L.^20^ Even considering that ceftriaxone is 85%–95% protein bound, the ceftriaxone level is still above the MIC for 100% of the dosing cycle.

This study has shown that an ofloxacin concentration of 4×MIC is rapidly bactericidal and has a prolonged PAE in time–kill experiments against *S.* Typhi isolates with intermediate ofloxacin susceptibility. In principle, higher doses of ofloxacin than used in these trials (>15 mg/kg/day) could be clinically effective against such infections, but may be limited by adverse effects, such as QT interval prolongation, tendonopathy and cardiac valve disorders. Low-dose regimens of ofloxacin (such as 200 mg twice daily in adults and 10–15 mg/kg/day in children used in these trials) risk selecting resistant *S.* Typhi and are best avoided. In contrast, ceftriaxone demonstrated slow bactericidal activity and an absent PAE against *S.* Typhi and this may contribute to the slow clinical responses seen with typhoid fever patients treated with ceftriaxone. With the emergence of isolates resistant to both ceftriaxone and fluoroquinolones the need to understand the activity of older antimicrobials and to study new agents against *S.* Typhi is critical.^3^

## Supplementary Material

dkab215_Supplementary_Data
