# Supplementary material for: Bactericidal activities and post-antibiotic effects of ofloxacin and ceftriaxone against drug-resistant Salmonella enterica serovar Typhi
Source: J Antimicrob Chemother. 2021 Jun 28;76(10):2606–9. doi: 10.1093/jac/dkab215 (PMC8446916; doi:10.1093/jac/dkab215)
Supplement: dkab215_Supplementary_Data [file dkab215_supplementary_data.docx]

# Supplementary data

**Supplementary Methods**

***Viable counting methods***

Samples for viable counts were taken immediately before the addition of the drug or control broth and then at 0.25, 0.5, 1, 2, 4, 6, 9, 12 and 24 hours after the addition of drug. Viable counts were performed in duplicate. Using an automatic pipette, 20μL of 3 dilutions, test broth or serial dilution in maximum recovery diluent (Oxoid), was spotted in duplicate onto well dried 20ml nutrient agar plates (Oxoid). For counts expected to be below 1,000 colony forming units (cfu/mL a sample of 100μl was taken directly from the broth and spread onto a well dried nutrient agar plate without dilution. For counts expected to be below 500 cfu/mL a 1ml sample was taken from the test broth and diluted in 9mls of Muller Hinton broth. Bacterial colonies were counted after 48 hours incubation at 35-37^0^C. At each time-point the average of the two counts for each sample was recorded for the dilution giving between 10 and 100 cfu per drop. Initial experiments were performed to ensure reproducibility of this counting method. A comparison of sampling with and without vortexing was performed to ensure that bacteria did not adhere to the glass universal bottles used for the dilutions. At each sampling time when all tests and controls had been sampled the bottles were returned immediately to the incubator. Each experiment was performed in triplicate.

# Table S1. Microbiology, treatment and outcome for 18 patients and isolates, MIC by agar incorporation, MBC by microbroth dilution

| Patient  code | Susceptibility pattern^1^ | Haplotype^2^ | GyrA mutations | Ofloxacin | | Ceftriaxone | | Ofloxacin treatment given and response | | | |
| --- | --- | --- | --- | --- | --- | --- | --- | --- | --- | --- | --- |
|  |  |  |  | MIC mg/L | MBC  mg/L | MIC mg/L | MBC  mg/L | Dose^3^ | Duration days | FCT (hrs)^4^ | Clinical progression |
|  |  |  |  |  |  |  |  |  |  |  |  |
| TY65 | FS | NA | NT | 0.06 | 0.12 | 0.06 | 0.50 | 15 | 2 | 84 | Uncomplicated recovery |
| TY73 | FS | Not-H58 | NT | 0.06 | 0.06 | 0.06 | 0.12 | 10 | 3 | 66 | Uncomplicated recovery |
| TY86 | FS | Not–H58 | NT | 0.06 | 0.06 | 0.06 | 0.12 | 10 | 3 | 66 | Uncomplicated recovery |
| TY98 | FS | Not–H58 | NT | 0.06 | 0.12 | 0.06 | 0.25 | 10 | 3 | 84 | Uncomplicated recovery |
| CT4 | FS | Not–H58 | NT | 0.06 | 0.25 | 0.03 | 0.12 | 15 | 3 | 84 | Uncomplicated recovery |
| CT66 | FS | H58 | NT | 0.06 | 0.12 | 0.12 | 0.50 | 15 | 3 | 78 | Uncomplicated recovery |
|  |  |  |  |  |  |  |  |  |  |  |  |
| TY77 | MDR NA^S^ | Not–H58 | NT | 0.06 | 0.12 | 0.12 | 0.25 | 15 | 2 | 60 | Uncomplicated recovery |
| TY84 | MDR NA^S^ | Not–H58 | NT | 0.03 | 0.12 | 0.06 | 0.25 | 15 | 2 | 60 | Uncomplicated recovery |
| TY90 | MDR NA^S^ | H58 | NT | 0.12 | 0.50 | 0.12 | 0.25 | 15 | 2 | 126 | Uncomplicated recovery |
| TY97 | MDR NA^S^ | H58 | NT | 0.06 | 0.25 | 0.12 | 0.25 | 15 | 2 | 48 | Uncomplicated recovery |
| CT55 | MDR NA^S^ | H58 | NT | 0.06 | 0.25 | 0.12 | 0.25 | 15 | 3 | 90 | Uncomplicated recovery |
| CT65 | MDR NA^S^ | H58 | NT | 0.06 | 0.12 | 0.12 | 0.50 | 15 | 3 | 108 | Uncomplicated recovery |
|  |  |  |  |  |  |  |  |  |  |  |  |
| TY62 | MDR NA^R^ | NA | NA | 0.12 | 0.25 | 0.06 | 0.25 | 10 | 3 | 264 | Rescue treatment with ofloxacin for 7days |
| TY169 | MDR NA^R^ | H58 | S83F (TCC-TTC) | 1.0 | 2.0 | 0.12 | 0.25 | 10 | 3 | 282 | Rescue treatment with ofloxacin for 7 days |
| CT30 | MDR NA^R^ | Not-H58 | D87G (GAC-GGC) | 0.25 | 0.5 | 0.12 | 0.12 | 15 | 2 | 294 | Slow recovery |
| CT31 | MDR NA^R^ | Not-H58 | D87G (GAC-GGC) | 0.25 | 0.50 | 0.12 | 0.25 | 15 | 3 | 204 | Slow recovery |
| CT75 | MDR NA^R^ | Not-H58 | D87G (GAC-GGC) | 0.50 | 1.0 | 0.06 | 0.25 | 15 | 2 | 144 | Uncomplicated recovery |
| CT76 | MDR NA^R^ | Not-H58 | D87G (GAC-GGC) | 0.50 | 0.50 | 0.06 | 0.12 | 15 | 3 | 366 | Rescue treatment with ceftriaxone for 7 days, repeat blood culture positive, gastrointestinal bleeding |

^1^ Susceptibility pattern: FS - susceptible to ampicillin, chloramphenicol, trimethoprim-sulphamethoxoazole, nalidixic acid and ceftriaxone with ofloxcain MIC ≤ 0.12 mg/L; MDR NA^S^ - resistant to; ampicillin, chloramphenicol and trimethoprim-sulphamethoxoazole, susceptible to nalidixic acid and ceftriaxone with ofloxcin MIC ≤ 0.12 mg/L; MDR NA^R^ resistant to; ampicillin, chloramphenicol and trimethoprim-sulphamethoxoazole, nalidixic acid and susceptible to ceftriaxone with ofloxacin MIC of 0.25-1.0 mg/L

^2^ Haplotye H58 or non-H58

^3^ Ofloxacin dose in mg/kg/day given in two divided doses

^4^ Fever clearance time - time since treatment began for the temperature to fall below 37.5^o^C and remain at or below 37.5^o^C for 48 hours

NA – isolate not available for this test; NT -not tested

^5^ Isolate TY62 was nalidixic acid resistant but had an ofloxacin MIC of 0.125 mg/L that is susceptible according to CLSI guidelines. The fever clearance time response with ofloxacin treatment was prolonged, in keeping with the other nalidixc acid resistant isolates. Unfortunately, this isolate was not available for determination of the H58 haplotype and the *gyrA* gene mutation.

**Figure S1.** Kaplan-Meier survival curve comparing the fever clearance times for patients infected with an isolate of *S.*Typhi fully susceptible, nalidixic acid susceptible (FS, n=6); an isolate of *S.*Typhi multidrug resistant and nalidixic acid susceptible (MDR NA^S^, n=6); an isolate of *S.*Typhi multidrug resistant and nalidixic acid nalidixic acid resistant (MDR NA^R^, n=6) and treated with ofloxacin at 10-15 mg/kg/day for two to three days.
